# A Novel KGD-Based αIIbβ3 Antagonist Prevents Arterial Thrombosis While Preserving Hemostasis and Avoiding Thrombocytopenia

**DOI:** 10.3390/ijms26104530

**Published:** 2025-05-09

**Authors:** Yu-Ju Kuo, Ching-Hu Chung, Chun-Chao Chen, Ju-Chi Liu, Kuan-Rau Chiou, Joen-Rong Sheu, Woei-Jer Chuang, Tur-Fu Huang

**Affiliations:** 1 Division of Cardiology, Department of Internal Medicine, Shuang Ho Hospital, Taipei Medical University, Taipei 23561, Taiwan; 23580@s.tmu.edu.tw (Y.-J.K.); b101092035@tmu.edu.tw (C.-C.C.); liumdcv@tmu.edu.tw (J.-C.L.); krchiou@hotmail.com (K.-R.C.); 2 Department of Medicine, Mackay Medical College, New Taipei City 252, Taiwan; chchung@mmc.edu.tw; 3 Division of Cardiology, Department of Internal Medicine, School of Medicine, College of Medicine, Taipei Medical University, Taipei 23561, Taiwan; 4 Graduate Institute of Medical Sciences, College of Medicine, Taipei Medical University, Taipei 11031, Taiwan; sheujr@tmu.edu.tw; 5 Department of Biochemistry, National Cheng Kung University Medical College, Tainan 701, Taiwan; 6 Graduate Institute of Pharmacology, College of Medicine, National Taiwan University, Taipei 100233, Taiwan

**Keywords:** integrin αIIbβ3, disintegrin, binding sites, bi-directional signaling, thrombosis, hemorrhagic risk

## Abstract

Current αIIbβ3 antagonists are potent antithrombotic agents, their clinical use is limited by the risk of life-threatening bleeding. Emerging evidence has highlighted key mechanistic differences between thrombosis and hemostasis, opening avenues for safer antithrombotic strategies. Targeting integrin αIIbβ3 outside-in signaling has been proposed to mitigate bleeding risk; however, the short half-life of peptide-based therapeutics remains a major challenge. In this study, we developed an optimized αIIbβ3 antagonist, KGDRR—a recombinant mutant protein derived from snake venom disintegrin, incorporating an Arg55 residue within the KGD loop—through systematic structure–activity relationship (SAR) analysis. Molecular docking revealed a critical cation–π interaction between Arg55 of KGDRR and Tyr122 of the β3 subunit, stabilizing integrin αIIbβ3 in an unliganded-closed conformation. Functionally, KGDRR selectively inhibited thrombus propagation by blocking ligand binding and downstream Gα13-mediated outside-in signaling while preserving initial thrombus core formation, which is a limitation of current αIIbβ3 inhibitors. Unlike conventional antagonists, KGDRR maintained αIIbβ3 in an unliganded-closed conformation without inducing the integrin activation and conformational change that lead to immune-mediated platelet clearance and thrombocytopenia. In animal models, KGDRR effectively suppressed thrombus growth without causing thrombocytopenia or prolonging bleeding time. Furthermore, intramuscular administration of KGDRR achieved a functional half-life 3.5 times longer than that of the clinically used antithrombotic eptifibatide at equivalent antithrombotic efficacy. In conclusion, KGDRR exhibits potent antithrombotic activity with a favorable safety profile and enhanced pharmacokinetic stability. These findings position KGDRR as a promising next generation αIIbβ3 antagonist with the potential to improve clinical outcomes in antithrombotic therapy.

## 1. Introduction

Cardiovascular disease remains the leading cause of death in the United States, surpassing cancer and chronic lung disease [1]. Intravascular thrombus formation is a major contributor to acute coronary syndromes, necessitating the use of antithrombotic therapies, including antiplatelet and anticoagulant agents, to reduce morbidity and mortality. In the context of percutaneous coronary interventions (PCI), one key strategy which has emerged involves targeting integrin αIIbβ3, with these antagonists playing a central role in preventing periprocedural thrombosis. Currently, three clinically approved αIIbβ3 antagonists—tirofiban, eptifibatide, and abciximab—are widely used due to their rapid onset and high efficacy [1]. However, their clinical utility is often limited by life-threatening thrombocytopenia and bleeding risks, particularly in PCI patients [2]. Given these challenges, there is an urgent need for safer αIIbβ3 antagonists that effectively prevent thrombosis without increasing bleeding risk.

Our research on snake venom disintegrins began with the identification of trigramin, the first Arg-Gly-Asp (RGD)-containing single polypeptide, which contributed to the development of eptifibatide [3,4]. To address the bleeding complications associated with αIIbβ3 antagonists, we subsequently isolated and characterized TMV-7, a natural αIIbβ3 antagonist from *Trimeresurus mucrosquamatus* snake venom. TMV-7 binds to the αIIb β-propeller domain without inducing conformational changes or significantly increasing bleeding risk at therapeutic doses. However, administration of extremely high doses of TMV-7—20 times the minimum effective level of TMV-7 and existing clinical antithrombotics—resulted in prolonged bleeding times in animal models [5], highlighting the challenge of balancing antithrombotic efficacy with hemostatic safety. While peptide-based αIIbβ3 antagonists have shown promising hemostatic safety and therapeutic potential, their clinical application is often hindered by a short half-life, limiting their clinical application [6].

In this study, we introduce KGDRR, a novel αIIbβ3 antagonist that demonstrates superior safety and efficacy compared to TMV-7 and existing clinical antithrombotics. KGDRR selectively inhibits integrin αIIbβ3 outside-in signaling without inducing conformational activation, effectively preventing thrombosis while preserving hemostasis. To evaluate its therapeutic potential, we assessed the antithrombotic activity, half-life, and bleeding risk of KGDRR in a pig model. Additionally, using rational site-directed mutagenesis, we modified specific amino acid residues flanking the essential R(K)GD sequence, focusing on the linker region, RGD motif, and C-terminal domain. We further explored the structure–activity relationship of these mutants through molecular docking and dynamic simulations with αIIbβ3 complexes. Molecular docking revealed a crucial cation-π interaction between Arg55 in KGDRR and Tyr122 of the β3 subunit, stabilizing αIIbβ3 in an unliganded-closed state.

Our findings provide critical insights into the molecular interactions between KGDRR and integrin αIIbβ3, contributing to a deeper understanding of thrombosis and hemostasis. By elucidating these fundamental interactions, this study may pave the way for the development of safer and more effective antithrombotic therapies.

## 2. Results

### 2.1. Structure-Activity Relationships in Optimizing TMV-7 Derivatives for Enhanced Antiplatelet Efficacy with Reduced Bleeding Risk

Current integrin antagonists function by blocking integrin ligand binding, but they often induce conformational changes that lead to adverse effects, such as thrombocytopenia and bleeding. In our previous study, we purified TMV-7, an RGD-containing disintegrin, which demonstrated an improved safety profile. TMV-7 neither induced conformational changes in αIIbβ3 nor primed resting platelets for ligand binding, contributing to its reduced bleeding tendency [5]. At efficacious antithrombotic doses, TMV-7 did not increase bleeding risk in vivo; however, when intravenously administered to mice at a high dose of 5 mg/kg (20 times the antithrombotic dose), it significantly prolonged tail bleeding times from 58.41 to 188.00 s (*p* < 0.05; Table 1).

To develop an optimal antithrombotic agent with an improved safety profile, we introduced mutations in the TMV-7 sequence, focusing on the RGD loop and C-terminal domain. We systematically evaluated these mutants for antiplatelet potency, safety index values (see the Methods section or our previous study [5] for detailed calculations), and tail bleeding times (Table 1).

An amino acid substitution in the RGD loop of TMV-7 from ^50^ARGDNP^55^ to ^50^ARGDWN^55^ or ^50^ARGDFP^55^ was previously found to increase bleeding risk [5]. In contrast, our study revealed that N55R (^50^ARGDWR^55^) or P55R (^50^ARGDFR^55^) mutations significantly reduced bleeding risk (Table 1). Based on these findings, we further modified the RGD loop of TMV-7 from ^50^ARGDNP^55^ to ^50^ARGDNR^55^, resulting in a 12-fold increase in the safety index compared to TMV-7.

To refine the structure–activity relationship (SAR), we performed alanine scanning mutagenesis of the RGD loop and identified that the N54A (^50^ARGDAR^55^) mutation led to a 160-fold increase in the safety index, suggesting a critical role for Arg55 in minimizing bleeding risk. Building on this insight, we modified the sequence from ^50^ARGDAR^55^ to ^50^ARGDRR^55^ to enhance antiplatelet potency while preserving safety. To further improve specificity for αIIbβ3, an arginine-to-lysine substitution (R51K) was introduced. The resulting ^50^AKGDRR^55^ mutant, derived from TMV-7 (originally ^50^ARGDNP^55^), demonstrated a greater than 143-fold increase in the safety index compared to the parental TMV-7. Based on these findings, we designated ^50^AKGDRR^55^ as the lead antithrombotic candidate and referred to it as “KGDRR (RR)” throughout the study. Here, “RR” denotes the specific KGDRR variant under investigation.

A similar phenomenon was observed in SAR studies of rhodostomin (Rn), a disintegrin with high antithrombotic potency but an increased bleeding risk [7]. Notably, modifying rhodostomin by replacing ^50^PRGDMP^55^ with ^50^ARGDRR^55^ resulted in a 1000-fold improvement in its safety index, reinforcing the essential role of Arg55 in stabilizing integrins in the resting state and minimizing bleeding risk.

### 2.2. Distinct Binding Characteristics of Pure and Partial Antagonists to Integrin αIIbβ3

Typically, partial antagonist binding to αIIbβ3 induces a conformational transition from a bent (inactive) to an open (active) form, triggering pathological signaling pathways that contribute to thrombocytopenia [8]. Theoretically, pure antagonists stabilize αIIbβ3 in its inactive conformation, preventing unintended platelet activation and minimizing bleeding risk.

**Figure 1:**
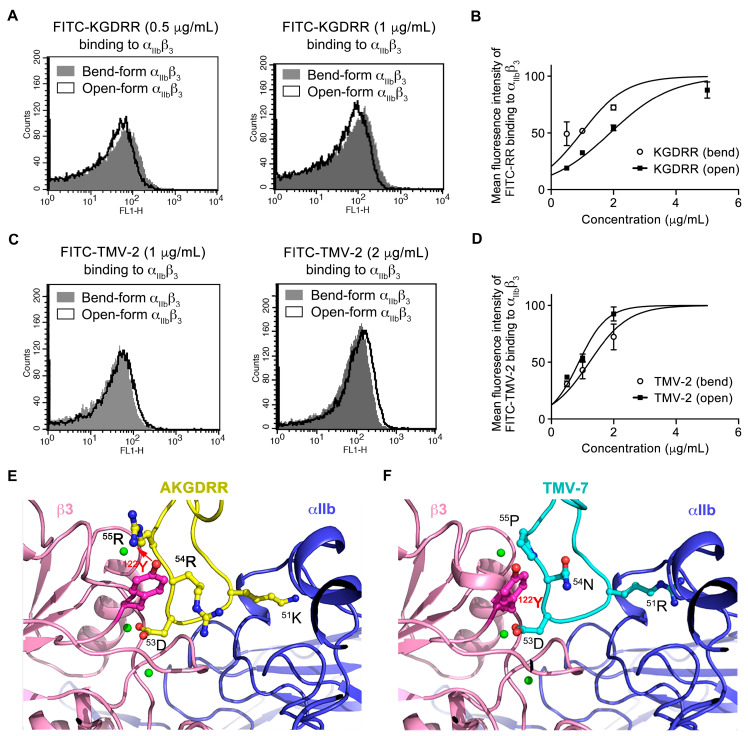
The different binding capacity of pure and partial antagonists on integrin α_IIb_β_3_ in bend-form or PAR-4-stimulated open-form, and the docking of pure antagonists at the whole surface of α_IIb_β_3_. (**A**–**D**) Human platelet suspension (PS) was incubated with PAR-4 100 μM (open-form) or without PAR-4 (bend-form) for 5 min, and probed by FITC-KGDRR (A, 0.5 and 1 μg/mL; B, increasing concentrations) or FITC-TMV-2 (C, 1 and 2 μg/mL; D, increasing concentrations) as indicated, subsequently analyzed by flow cytometry. (*n* = 5; Data presented as mean ± SEM) (**E**,**F**) The docking of TMV-7 (^50^ARGDNP^55^) and its ^50^AKGDRR^55^ mutant into integrin α_IIb_β_3_. Ribbon diagrams show the key contact surface between integrin αIIbβ3 and the KGDRR loop (**E**) or TMV-7 (**F**). The propeller domain of the αIIb subunit and the βA domain of the β_3_ subunit are shown in purple and pink, respectively. The interacting residues are shown in the ball-and-stick representation. The Mn^2+^ ion of the β_3_ subunit formed hydrogen bonds with the carboxylate oxygen of the RGD residue. At the center of the KGDRR–βA contact was a cation-π edge-to-face interaction (red arrow) of the mutant Arg55 in KGDRR with Tyr122 side chains in the α1 helix of βA. Note that the inward movement of Tyr122 (light pink) in TMV-7-bound βA would clash with the Pro55 side chain.

To investigate the conformational preference of KGDRR, we incubated human platelets with PAR-4 (100 μM) to induce the open form of αIIbβ3 or left them untreated to maintain the bent form. Binding was assessed using FITC-labeled KGDRR, and its binding characteristics were compared with those of TMV-2, a partial antagonist known for its low safety index and high bleeding tendency in vivo (Table 1). The results demonstrated that KGDRR preferentially binds to the bent, inactive form of αIIbβ3 in a concentration-dependent manner (Figure 1C,D), whereas TMV-2 exhibits a strong preference for the liganded-open form of αIIbβ3 (Figure 1E,F).

Based on their safety profiles, all disintegrins purified by our team were classified into two functional categories. Disintegrins with a high safety profile, termed “pure antagonists”, tend to bind to the bent (inactive) form of αIIbβ3, preserving its original conformation and preventing integrin-induced conformational changes, thereby maintaining physiological hemostasis. Representative examples include TMV-7 [5], its derivative TMV-7 based-^50^AKGDRR^55^ mutant, and Rhodostomin based-^50^ARGDRR^55^ mutant. In contrast, disintegrins classified as “partial agonists” preferentially bind to the liganded-open form of αIIbβ3, inducing conformational changes that not only enhance antithrombotic effects but also lead to significant bleeding risks. Examples include TMV-2 [5], Trigramin [4], Eptifibatide [9], and Rhodostomin [10].

### 2.3. Structural Basis of KGDRR’s Enhanced Safety Profile and Unique Binding Mechanism to Integrin αIIbβ3

Although both TMV-7 and its derivative KGDRR function as pure antagonists, KGDRR (^50^AKGDRR^55^), which carries N54R and P55R mutations from TMV-7 (^50^ARGDNP^55^), exhibited a 160-fold increase in the safety index (Table 1). This dramatic improvement indicates that Arg55 plays a critical role in minimizing bleeding risk. To further elucidate the effects of Arg54 and Arg55 in KGDRR on the intrinsic binding mechanism and conformational stability of αIIbβ3, we performed structural modeling and compared KGDRR with TMV-7.

Using X-ray crystal structures of integrin αIIbβ3 in complex with a GRGDSP peptide (PDB code: 3ZE2 [11]) and CCP4i 7.1 software, we generated models of the αIIbβ3–TMV-7 and αIIbβ3–KGDRR complexes (Figure 1E,F). The KGDRR–αIIbβ3 complex revealed a central cation-π interaction between Arg55 in the RGD-containing loop of KGDRR and Tyr122 of the β3 subunit (Figure 1A, Table 2). This interaction was abolished when the RGD domain of KGDRR (^50^AKGDRR^55^) was replaced with that of (^50^ARGDNP^55^), underscoring the critical role of Arg55 in stabilizing the inactive conformation of αIIbβ3.

Our findings highlight that the Arg55-Tyr122 cation-π interaction is the key structural feature responsible for reducing the bleeding tendency of KGDRR. Previous studies have shown that in barbourin and eptifibatide, two partial agonists of αIIbβ3 associated with a high bleeding risk, their tryptophan side chains point inward toward the center of the RGD loop and away from the Tyr122 side chain of the βA domain [12], thereby preventing the formation of a central cation-π interaction.

Structural analysis of the KGDRR–αIIbβ3 complex provides a mechanistic explanation for the unique behavior of KGDRR as a pure antagonist despite its canonical KGD motif. Collectively, our structural and mutational studies suggest that the Arg55–Tyr122 cation-π interaction immobilizes αIIbβ3 in a bent, inactive conformation, thereby inhibiting ligand binding through a distinct mechanism. These findings not only enhance our understanding of integrin αIIbβ3 regulation but also provide valuable insights for the future development of safer αIIbβ3 antagonists with improved therapeutic profiles.

### 2.4. Pure Antagonists Do Not Induce Conformational Changes or Exposure of the Ligand-Induced Binding Site (LIBS) in Integrin αIIbβ3

**Figure 2:**
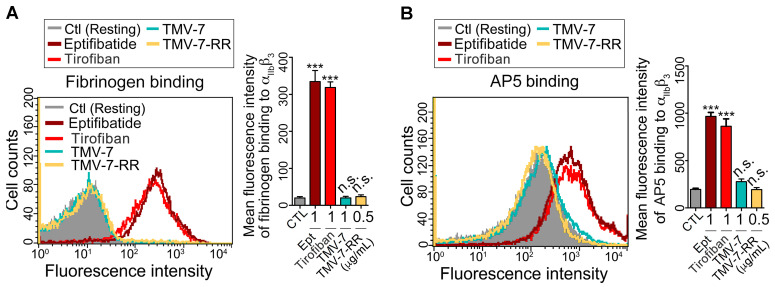
The effects of partial agonists and pure antagonists on the priming effect and ligation-induced LIBS exposure. (**A**) Partial agonists (i.e., eptifibatide (Ept) and Tirofiban) or pure antagonists (i.e., TMV-7 and KGDRR) were incubated with PS for 30 min. After washing, PS was labeled with Alexa 488-conjugated fibrinogen (200 μg/mL) for 30 min, then analyzed by flow cytometry. (*n* = 5) (**B**) PS was incubated with agents for 30 min, and then probed with 20 μg/mL LIBS-specific AP5 mAb and analyzed by flow cytometry. The data shown in the right panel is the mean fluorescence intensity of platelets in the presence of each agent. (*n* = 7) (Data presented as mean ± SEM; Non-significance (n.s.), *** *p* < 0.001 as compared with CTL group by Dunnett’s test).

Previous studies have shown that RGD-mimetic drugs, such as eptifibatide and tirofiban, induce conformational changes in αIIbβ3, leading to immune-mediated platelet clearance and thrombocytopenia [13,14]. To further evaluate whether the antagonists studied in this work trigger conformational changes, we examined their ability to “prime” resting platelet αIIbβ3 for fibrinogen binding [15]. As shown in Figure 2A, treatment with partial agonists (eptifibatide and Tirofiban) significantly promoted fibrinogen binding, indicating their propensity to induce conformational changes in αIIbβ3. In contrast, treatment with pure antagonists (TMV-7 and KGDRR) did not induce fibrinogen binding to resting platelets, suggesting that these compounds have minimal intrinsic ability to alter the conformational state of αIIbβ3.

Exposure of ligand-induced binding sites (LIBS) serves as a marker for αIIbβ3 conformational changes [15]. Consistent with the fibrinogen binding results, Figure 2B demonstrates that eptifibatide and Tirofiban significantly increased LIBS-specific monoclonal antibody (mAb) AP5 binding to platelets, confirming that these partial agonists trigger integrin conformational changes, leading to LIBS exposure. In contrast, pure antagonists (TMV-7 and KGDRR) did not induce LIBS exposure, further supporting their superior safety profile and lack of unintended integrin activation. These findings highlight the distinct binding properties of pure antagonists, reinforcing their potential as safer alternatives to current αIIbβ3 inhibitors.

### 2.5. Molecular Mechanisms of Pure Antagonists in Human Platelets

**Figure 3:**
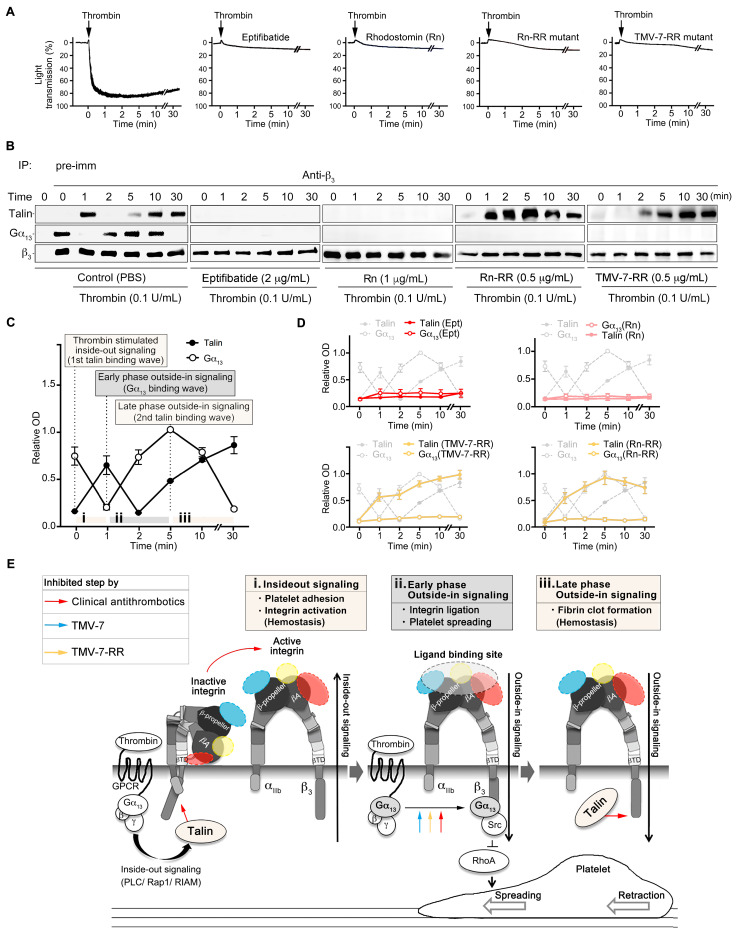
Effect of the partial agonists and pure antagonists on the mutually exclusive binding of talin and Gα_13_ to cytoplasmic β_3_ of integrin α_IIb_β_3_ in human platelets. (**A**–**D**), Human PS was pretreated with PBS (Control) and agents in the presence of thrombin, and then solubilized at various time points. Typical turbidity changes (**A**) in human PS indicate platelet aggregation. The platelets were lysed at the indicated time points and immunoprecipitated with anti-β_3_ (**B**), consequently immunoblotted for Gα_13_, talin, and β_3_. Quantification of immunoblots OD, optical density (**C**,**D**). In the control group (**C**), physiological processes of integrin bidirectional signaling were divided into three stages (i: the first talin binding wave, ii: Gα_13_ binding wave, iii: the second talin binding wave), driven by talin or Gα_13_, respectively. (Data presented as mean ± SD; *n* = 6) (**E**), A hypothetical model for the selectively inhibitory effect of KGDRR on integrin bidirectional signaling and related physiological activity. TMV-7 and KGDRR selectively inhibit the Gα_13_-mediated early phase of outside-in signaling without affecting the first- and the second talin-binding wave through distinct binding mechanisms. Arrows indicate steps that are inhibited.

To further investigate the molecular mechanisms by which pure antagonists inhibit platelet aggregation without inducing bleeding, we examined their effects on the mutually exclusive binding of talin and Gα13 to the β3 cytoplasmic domain. Immunoprecipitation was performed on lysates from thrombin-treated human platelets using anti-β3 antibodies, followed by immunoblotting for Gα13, talin, and β3 (Figure 3A,B). Talin and Gα13 play essential roles in thrombin-induced integrin signaling, with Gα13 mediating outside-in signaling, which regulates platelet spreading and thrombus stabilization, while talin is responsible for inside-out signaling, leading to αIIbβ3 activation and primary hemostasis [6].

In the control group treated with PBS, talin–β3 association exhibited a biphasic pattern (Figure 3B, left panels). The first wave (Figure 3C(i)) of talin association with the β3 cytoplasmic tail was observed upon thrombin stimulation through inside-out signaling, occurring before integrin ligation (Figure 3C(ii)). The second wave (Figure 3C(iii)) of talin–β3 interaction emerged after complete platelet aggregation (Figure 3C(ii)). In contrast, the Gα13–β3 interaction followed an inverse pattern, decreasing during thrombin-stimulated inside-out signaling (Figure 3C(i)), increasing after integrin ligation when the first talin-binding wave diminished (Figure 3C(ii)), and subsequently decreasing again during the second talin-binding wave (Figure 3C(iii)). These two waves of talin binding are critical for primary hemostasis and mediate key physiological outcomes of integrin activation [16], including αIIbβ3 activation (Figure 3E(i)) [17] and fibrin clot formation (Figure 3E(iii)) [18].

Interestingly, we observed that partial agonists, including eptifibatide and Rhodostomin, significantly inhibited talin binding to β3, suggesting their potential to disrupt integrin-mediated signaling (Figure 3D). In contrast, talin localization to cytosolic β3 remained unaffected by the pure antagonists TMV-7, its derivative KGDRR, and the Rhodostomin-RGDRR mutant (Figure 3B,D). Notably, structural modifications—either replacing ^50^ARGDNP^55^ with ^50^AKGDRR^55^ in TMV-7 or substituting ^50^PRGDMP^55^ with ^50^ARGDRR^55^ in Rhodostomin—successfully preserved talin–β3 interactions, which are crucial for primary hemostasis.

Structural analysis of the KGDRR–αIIbβ3 and Rhodostomin-RGDRR–αIIbβ3 complexes revealed a central cation-π interaction between Arg55 in the RGD-containing loop of disintegrins and Tyr122 of the β3 subunit. This Arg55-Tyr122 cation-π interaction appears to be the key structural feature responsible for stabilizing integrins in their inactive conformation, thereby preventing conformation-induced thrombocytopenia. Further supporting this observation, in vivo bleeding time data demonstrated that these modifications led to a 1000-fold improvement in the safety index (Table 1).

These findings underscore the critical role of Arg55 in stabilizing integrins in their resting state and maintaining their inactive conformation, effectively minimizing bleeding risk while preserving essential hemostatic function. Moreover, they demonstrate that pure antagonists KGDRR and Rhodostomin-RGDRR, both containing Arg55, act as selective inhibitors of Gα13-mediated outside-in signaling while preserving talin–β3 interactions, a mechanism essential for αIIbβ3-dependent primary hemostasis. This selective inhibition further highlights their potential as safer antiplatelet agents, particularly when compared with partial agonists, which disrupt both outside-in and inside-out signaling, leading to an increased bleeding risk.

### 2.6. Both Partial Agonists and Pure Antagonists Inhibit Agonist-Induced Integrin αIIbβ3 Ligation and Platelet Aggregation

**Figure 4:**
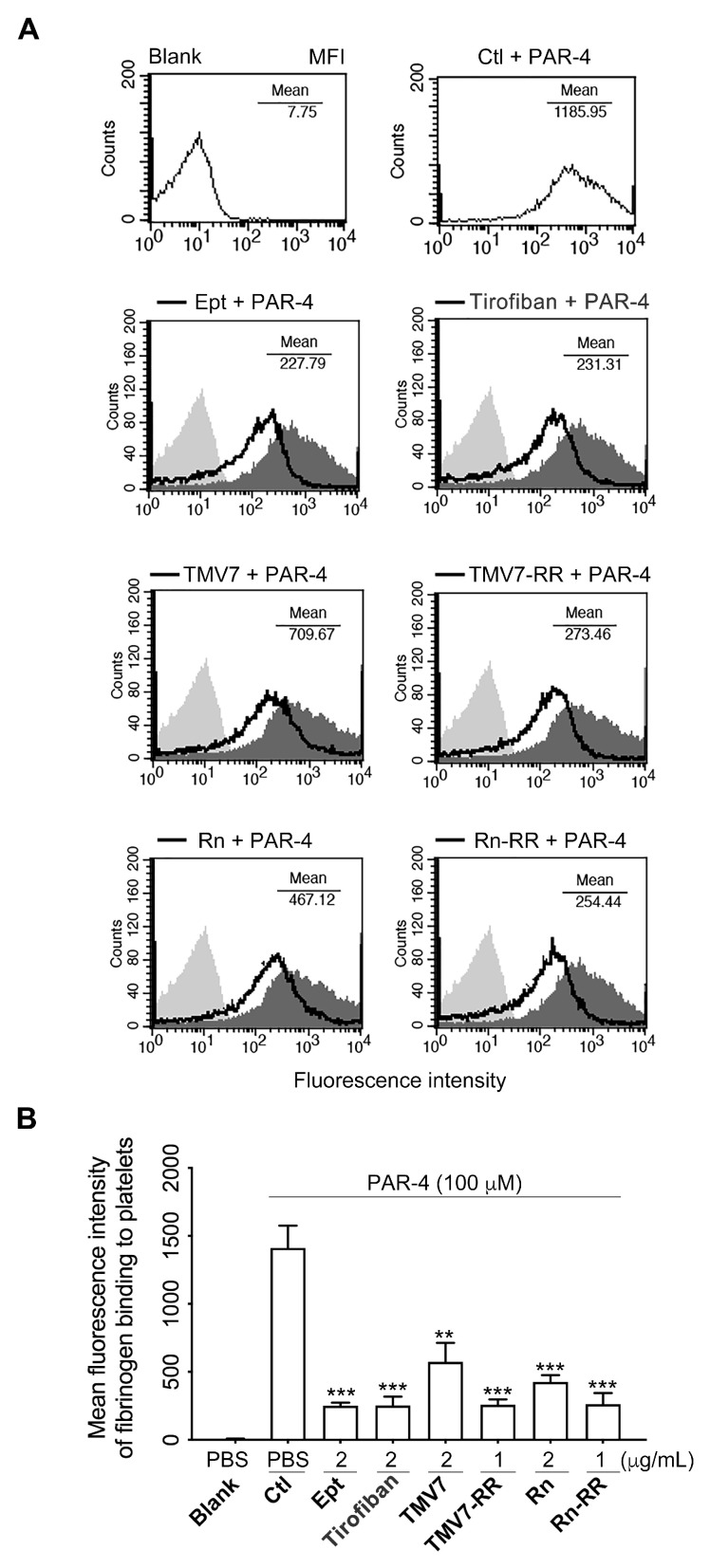
Effect of KGDRR on PAR-4-induced fibrinogen binding to the integrin α_IIb_β_3_. (**A**) Flow cytometric analysis of 100 μM PAR-4-induced fibrinogen binding to platelets pretreated with PBS, eptifibatide (Ept, 2 μg/mL), Tirofiban (2 μg/mL), TMV-7 (2 μg/mL), or TMV-7 based KGDRR mutant (TMV-7-RR, 1 μg/mL), Rhodostomin (Rn, 2 μg/mL), or Rhodostomin based KGDRR mutant (Rn-RR, 1 μg/mL) as indicated for 30 min. Resting platelets were used as a negative control. The histogram was representative of three similar experiments. (**B**) Data presented as mean ± SEM; Non-significance (n.s.), ** *p* < 0.01, and *** *p* < 0.001 as compared with PAR-4-activated group by Dunnett’s test).

Consistent with the co-immunoprecipitation results, which showed that all tested αIIbβ3 antagonists inhibit the ligand occupancy-induced switch from the talin-bound to the Gα13-bound state, flow cytometry analysis confirmed that these antagonists effectively blocked fibrinogen binding to PAR-4-stimulated platelets (Figure 4). This finding indicates that the pure antagonists KGDRR and Rn-RGDRR, both acting as selective inhibitors of Gα13-mediated outside-in signaling, effectively inhibited agonist-induced ligand binding to integrin αIIbβ3, thereby suppressing platelet aggregation.

To further evaluate their efficacy, we tested their ability to inhibit platelet aggregation induced by multiple agonists, including adenosine 5’-diphosphate (ADP), collagen, and U46619 (Table 3). As shown in Table 3, in human platelet-rich plasma (PRP), both TMV-7 and KGDRR exhibited concentration-dependent inhibition of platelet aggregation induced by ADP (20 μM), collagen (10 μg/mL), and U46619 (1 μM). Similarly, in human platelet suspension (PS), TMV-7 and KGDRR blocked platelet aggregation triggered by thrombin (0.1 U), collagen (10 μg/mL), and U46619 (1 μM) in a dose-dependent manner.

These findings demonstrate that the pure antagonist KGDRR effectively inhibits platelet aggregation by specifically blocking fibrinogen binding to αIIbβ3 on agonist-activated platelets, a mechanism that represents a critical and common final step in platelet aggregation. This targeted inhibition highlights its potential as an effective antithrombotic and safe agent compared with existing αIIbβ3 antagonists.

### 2.7. The Pure Antagonist KGDRR Exhibits a Prolonged Half-Life, Favorable Safety Profile, and Potent Antithrombotic Activity in Murine Models

**Figure 5:**
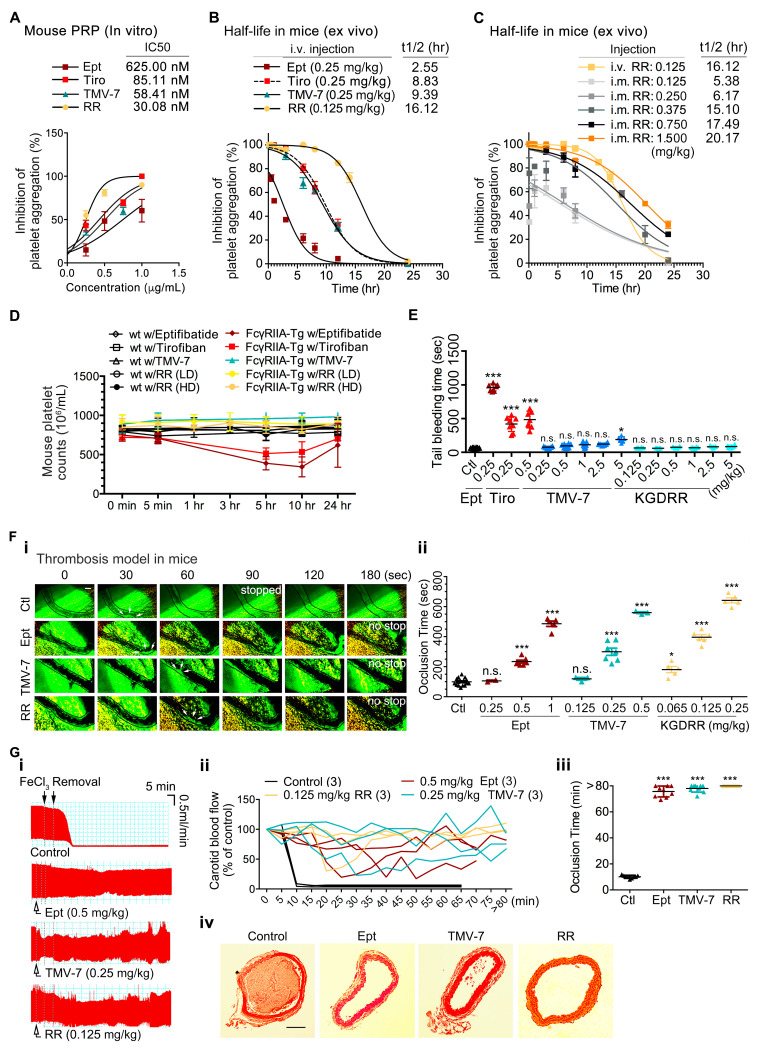
Effect of KGDRR on ex vivo platelet aggregation and in vivo thrombus formation in murine models. (**A**) In vitro platelet aggregation assay in mouse PRP treated with saline (control) or increasing concentrations of Eptifibatide (Ept), Tirofiban (Tiro), TMV-7, or KGDRR (RR) in response to 10 μg/mL collagen. The IC_50_ values for platelet aggregation inhibition are shown. (**B**) Functional half-life calculations of intravenous treatments in inhibiting ex vivo platelet aggregation in mouse PRP. Mice received intravenous injections of Eptifibatide (Ept), Tirofiban (Tiro), TMV-7, or KGDRR (RR) at increasing doses. Blood samples were collected at the indicated time points (0.83, 1, 3, 6, 9, and 24 h) post-injection. PRP was isolated by centrifugation, and collagen (10 μg/mL) was added to trigger platelet aggregation. (**C**) Functional half-life of intramuscularly administered KGDRR (RR) in inhibiting ex vivo platelet aggregation in mouse PRP. Mice were intramuscularly injected with increasing doses of KGDRR, and blood samples were collected at the specified time intervals. PRP was obtained via centrifugation, followed by collagen (10 μg/mL) stimulation to assess platelet aggregation. (**D**) Assessment of immune thrombocytopenia in an FcγRIIa-transgenic mouse model. Wild-type and FcγRIIa-transgenic mice received intravenous injections of Eptifibatide (0.5 mg/kg), Tirofiban (0.025 mg/kg), TMV-7 (0.25 mg/kg), or KGDRR at low-dose (LD, 0.125 mg/kg) or high-dose (HD, 2.5 mg/kg). Platelet counts were measured before and at specified time points following treatment. (**E**) Tail bleeding time measurement in FcγRIIa-transgenic mice, recorded 5 min post-intravenous injection of the respective agents. The mean bleeding time is indicated as (—), and each symbol represents an individual mouse (*n* ≥ 3 per group). Data are presented as mean ± SEM. Statistical significance was determined using Dunnett’s test, with n.s. (not significant), * *p* < 0.05, and *** *p* < 0.001 compared to the control group. (**F**) Effect of agents on illumination-induced mesenteric venous thrombosis in mice. Representative images of the bright-field microvascular histology (**F**(**i**)) showing the progression of fluorescent dye-induced platelet-rich thrombus formation in mouse mesenteric venules at the indicated time points (Scale Bar, 50 μm). Arrows indicate the thrombi which were first observed. Each different symbol (**F**(**ii**)) represents the occlusion time of the individual mouse. (**G)** Effect of agents on FeCl_3_-induced mouse carotid artery thrombosis. Typical arterial blood flow charts (**G**(**i**)), the time-dependent blood flow curve (**G**(**ii**)), and the occlusion time (**G**(**iii**)) of FeCl_3_-induced occlusive thrombosis of carotid artery in the individual mouse are shown. The histologic section (**G**(**iv**)) of FeCl_3_-treated carotid artery. All panels are ×100 with the same scale bar in left panel (Scale Bar, 100 μm). (*n* ≥ 3 in each group; data presented as mean ± SEM; Non-significance (n.s.), * *p* < 0.05 and *** *p* < 0.001 as compared with the saline-treated group by Dunnett’s test).

To evaluate the antithrombotic efficacy and pharmacokinetic profile of KGDRR, we assessed its activity in murine models. In vitro, KGDRR demonstrated greater potency in inhibiting collagen-induced platelet aggregation compared to eptifibatide in mouse PRP (Figure 5A). Ex vivo experiments revealed that, at equally efficacious doses, KGDRR exhibited a functional half-life that was 6.4 times longer than eptifibatide in mice (Figure 5B). Additionally, KGDRR displayed a stable pharmacokinetic profile following intramuscular administration (Figure 5C). The inhibitory effect of KGDRR on platelet aggregation persisted for over 40 h, with a half-life (t_1_/_2_) of 20.17 h at 1.5 mg/kg, highlighting its extended duration of action.

To further assess the safety profile of KGDRR, we compared its effects on platelet counts and hemostatic function with eptifibatide using an FcγRIIa-transgenic mouse model (Figure 5D,E). At efficacious antithrombotic doses, eptifibatide and tirofiban caused a significant reduction in platelet counts within 5 h in transgenic mice (Figure 5D). Additionally, in eptifibatide-treated mice (Figure 5E), bleeding times were significantly prolonged, coinciding with a time-dependent decrease in platelet counts.

In the mouse thrombosis model, intravascular administration of KGDRR effectively suppressed occlusive thrombosis in illuminated vessels, demonstrating greater efficacy than eptifibatide (Figure 5F(i,ii)). Further assessments in the FeCl_3_-induced carotid artery thrombosis model showed that, in untreated mice, vessel occlusion occurred within 10 min following FeCl_3_ injury, as illustrated by blood flow monitoring (Figure 5G(i,ii)). However, administration of eptifibatide, TMV-7, or KGDRR delayed occlusion times by over 80 min (Figure 5G(ii,iii)). Histological analysis of injured carotid arteries further confirmed that thrombus formation was significantly reduced following treatment with KGDRR, TMV-7, or eptifibatide compared to untreated controls (Figure 5G(iv)). These findings provide direct evidence that KGDRR, exhibits potent antiplatelet and antithrombotic activities in vivo.

From a clinical perspective, the extended half-life and potent antithrombotic efficacy of KGDRR, particularly when administered via intramuscular injection, offer significant practical advantages. The ability to maintain stable platelet inhibition over an extended period provides greater convenience for pre-hospital management of acute myocardial infarction and thrombotic stroke while also reducing overall healthcare costs compared to standard intravenous (IV) infusion therapies.

### 2.8. The Pure Antagonist KGDRR Exhibits Potent Antiplatelet Activity, a Prolonged Half-Life, and a Favorable Safety Profile in Swine Models

**Figure 6:**
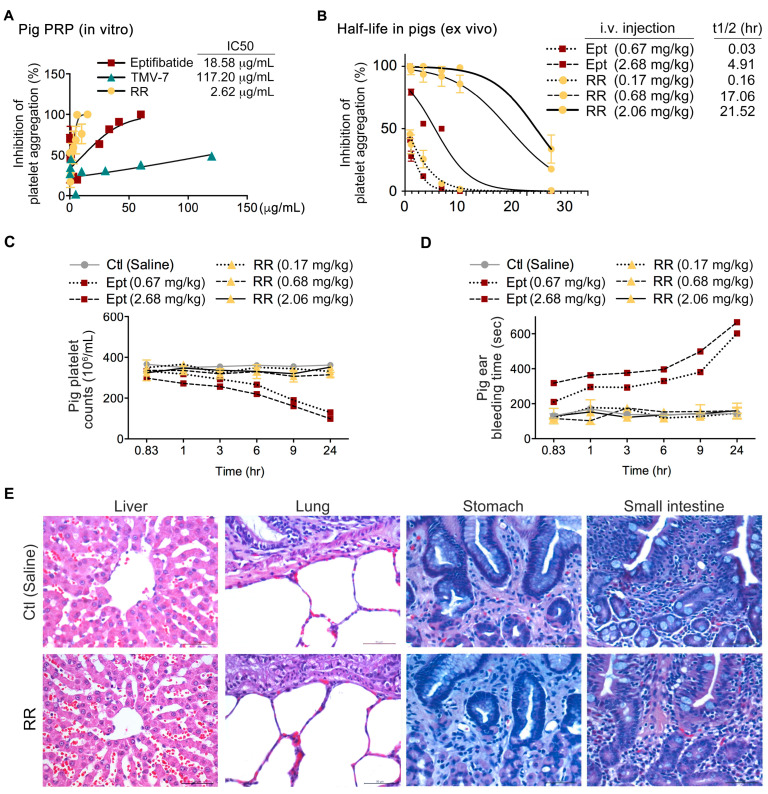
Effect of KGDRR on ex vivo platelet aggregation and in vivo hemostatic function in swine models. (**A**) In vitro platelet aggregation assay in pig PRP treated with saline (control) or increasing concentrations of eptifibatide, TMV-7, or KGDRR in response to 10 μg/mL collagen. The IC_50_ values for platelet aggregation inhibition are shown. (**B**) Functional half-life calculations of intravenous treatments in inhibiting ex vivo platelet aggregation in pig PRP. Pigs received intravenous injections of eptifibatide and KGDRR at increasing doses. Blood samples were collected at specified time intervals (0.83, 1, 3, 6, 9, and 24 h) post-injection. PRP was obtained via centrifugation, followed by collagen (10 μg/mL) stimulation to assess platelet aggregation. (**C**) Time-dependent effects of KGDRR and eptifibatide on thrombocytopenia in pigs. Whole blood samples were collected from pigs at 0.83, 1, 3, 6, 9, and 24 h post-intravenous injection, and platelet counts were measured at each time point to evaluate the impact of the treatments on thrombocytopenia. (**D**) Bleeding time measurement in pigs at 0.83, 6, 9, and 24 h post-administration of the agents. At each time point, the mean values of independent measurements were recorded as the bleeding time. (**E**) Representative histological sections of pig liver, lung, stomach, and small intestine specimens stained with hematoxylin and eosin (H&E) to assess potential tissue damage or bleeding complications (Scale bars, 50 μm). (*n* ≥ 3 per group; data are presented as mean ± SEM).

Given the limited clinical relevance of murine models in predicting pharmacokinetic and safety profiles in humans, and recognizing that swine models more closely resemble human physiology than rodent models, we further evaluated KGDRR in swine models to enhance the translational potential of our findings. In vitro, KGDRR demonstrated greater potency in inhibiting collagen-induced platelet aggregation compared to eptifibatide in porcine PRP (Figure 6A). Ex vivo, at equally efficacious doses, KGDRR exhibited a functional half-life that was 3.5 times longer than eptifibatide in pigs (Figure 6B).

To assess the in vivo safety profile of KGDRR, we compared its effects on platelet counts and hemostatic function with eptifibatide in swine models (Figure 6C,D). At therapeutically effective doses, eptifibatide caused a significant reduction in platelet counts within 10 h (Figure 6C). Additionally, in eptifibatide-treated swine (Figure 6D), bleeding times were significantly prolonged, coinciding with a time-dependent decrease in platelet counts. In contrast, KGDRR did not induce thrombocytopenia or prolong bleeding times at therapeutic doses, highlighting its superior safety profile.

Given the fatal gastrointestinal and retroperitoneal bleeding complications reported in patients receiving current αIIbβ3 antagonists in Phase III clinical trials (PURSUIT, ESPRIT, and IMPACT II), we further examined liver, lung, stomach, and small intestine tissues in pigs 24 h after intravenous administration of KGDRR (2.06 mg/kg) (Figure 6E). Histopathological analysis revealed no significant bleeding symptoms or tissue damage in KGDRR-treated pigs, compared to saline-treated controls.

These findings highlight KGDRR’s potential as a highly effective and safer antithrombotic agent compared to existing αIIbβ3 antagonists, ensuring an optimal balance between potent antithrombotic activity and physiological hemostasis.

### 2.9. Pure Antagonists Preserve Physiological Hemostasis in Human Whole Blood

**Figure 7:**
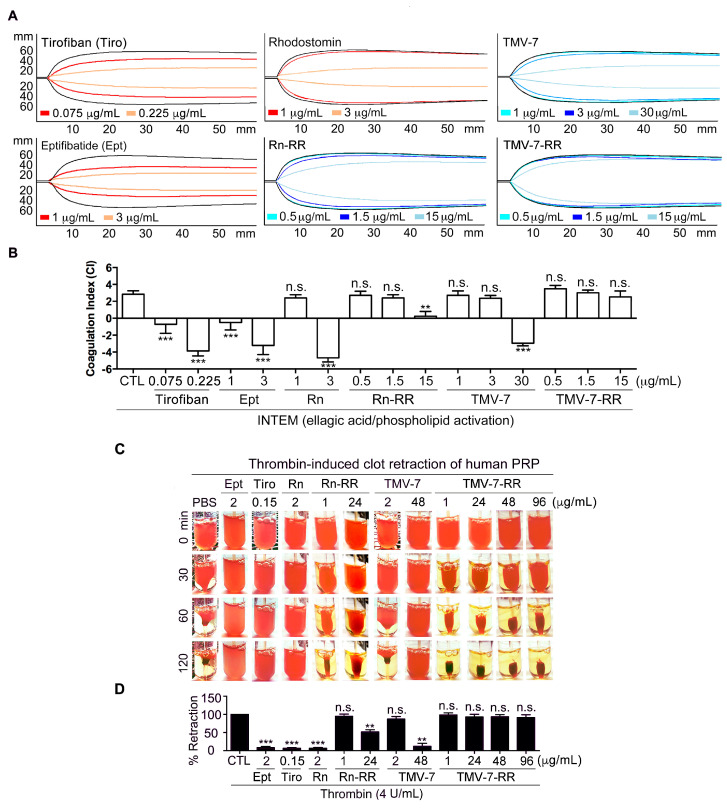
In vitro effect of pure antagonist KGDRR on the physiological hemostatic function in human whole blood. (**A**,**B**) Effect of antithrombotic agents on the hemostatic function by ROTEM in human whole blood. ROTEM trace (**A**) showed the relative activity of increasing concentrations of agents on coagulation indices of human whole blood. CTL (black line): in the absence of agent administration. Coagulation index (**B**) was determined for clot kinetics and the coagulation process was assessed (*n* = 5). (**C**,**D**) Human PRP was incubated with indicated agents for 3 min before the addition of thrombin (4 U/mL). The clot retraction (c) images were recorded every 30 min until 120 min. The serum volume of each tube (**D**) was measured after 120 min and compared with that of a control. (*n* = 5) (Data presented as mean ± SEM; Non-significance (n.s.), ** *p* < 0.01, and *** *p* < 0.001 as compared with resting group (**A**,**B**) or agonist-activated group (**C**,**D**) by Dunnett’s test.

Rotational thromboelastometry (ROTEM) is a clinically validated tool used to assess bleeding risk, particularly in perioperative and anesthetic settings, by monitoring drug effects on hemostasis [19,20]. To more precisely determine whether αIIbβ3 antagonists affect normal hemostatic function in humans, we evaluated their effects using ROTEM assays (Figure 7A,B). This method measures clotting time, clot formation time, α-angle, and maximum clot firmness, which together form the coagulation index (CI)—a key indicator of primary hemostasis.

To compare pure antagonists with clinical antithrombotics, we treated human whole blood with Tirofiban (0.075 μg/mL), Eptifibatide (1 μg/mL), and Rhodostomin (1 μg/mL) at their effective doses. These were compared with the pure antagonists TMV-7 (1 μg/mL), its KGDRR mutant (TMV-7-RR, 0.5 μg/mL), and Rhodostomin-KGDRR mutant (Rn-RR, 0.5 μg/mL) at equivalent effective doses. To further evaluate their safety profiles, the doses were increased threefold.

In human whole blood, Tirofiban, Eptifibatide, and Rhodostomin induced concentration-dependent suppression of all four ROTEM parameters, leading to a marked reduction in CI, indicative of a hypocoagulation state. In contrast, TMV-7, TMV-7-RR, and Rn-RR, at equally potent concentrations, had minimal effects on these parameters, suggesting a lower bleeding risk associated with pure antagonists. However, at 30 times the effective dose, TMV-7 (30 μg/mL) caused a marked reduction in CI. Notably, even at 30 times the effective dose, KGDRR mutant (15 μg/mL) and Rn-RR mutant (15 μg/mL) did not alter primary hemostasis indicators, demonstrating a wider safety margin compared to clinical antithrombotics like Tirofiban and Eptifibatide, as well as their original prototypes. This result indicates that KGDRR and Rn-RR containing Arg55 in the RGD loop preserve essential hemostatic function, underscoring the critical role of Arg55 in minimizing bleeding risk.

### 2.10. Thrombin-Induced Clot Retraction Confirms Superior Safety Profile of Pure Antagonists

To further assess the safety profile of pure antagonists, we performed a thrombin-induced clot retraction assay. Consistent with the ROTEM assay findings, thrombin-induced clot retraction in human PRP was significantly impaired by Eptifibatide (2 μg/mL) and Tirofiban (0.15 μg/mL), but remained unaffected by Rn-RR (1 μg/mL), TMV-7 (2 μg/mL), or KGDRR (1 μg/mL) (Figure 7C,D).

To further evaluate the safety margins of these pure antagonists, we tested them at 24-fold increased doses. At high concentrations (48 μg/mL), TMV-7 significantly impaired thrombin-induced clot retraction, indicating hemostatic disruption at supratherapeutic levels. However, KGDRR, even at 24 μg/mL and up to 96 μg/mL, maintained normal clot retraction, confirming that it does not interfere with integrin-dependent hemostatic mechanisms.

These results indicate that partial agonists, such as Tirofiban, Eptifibatide, and Rhodostomin, disrupt normal clot formation and platelet contraction, which are critical processes for primary hemostasis. In contrast, pure antagonists Rn-RR and KGDRR did not interfere with normal clot formation, further supporting their favorable safety profiles.

## 3. Discussion

In this study, we elucidated the molecular mechanisms underlying KGDRR’s superior safety profile through molecular docking analyses. By substituting the RGD domain of TMV-7 (^50^ARGDNP^55^) with ^50^AKGDRR^55^ or replacing that of Rhodostomin (^50^PRGDMP^55^) with ^50^ARGDRR^55^, we identified docking configurations that significantly enhanced safety—160-fold and 1000-fold, respectively. This subtle yet critical structural modification, particularly within six key residues of the RGD loop and C-terminal sequence, markedly increased specificity for αIIbβ3. Our structure–activity relationship studies and molecular docking analyses further suggest that the Arg55–Tyr122 cation-π interaction plays a crucial role in stabilizing αIIbβ3 in a bent, unliganded conformation. Notably, Tyr122 in the β3 domain is highly conserved across β1 and β2 integrins, reinforcing its pivotal role in integrin activation [21]. Rn-RGDRR and KGDRR, both containing Arg55 in the RGD loop, interact with Tyr122 in the integrin βA domain, forming a central cation-π interaction. This unique interaction stabilizes αIIbβ3 in its inactive conformation, effectively preventing ligand binding and undesired platelet activation.

Supporting these findings, KGDRR did not induce ligand-induced binding site (LIBS) exposure or increase LIBS-specific monoclonal antibody (mAb) AP5 binding to platelets, a known marker of αIIbβ3 conformational changes. This indicates that, upon binding to αIIbβ3, KGDRR does not induce integrin activation. Therefore, upon thrombin stimulation, KGDRR effectively prevented ligand binding to the bent, inactive conformation of αIIbβ3, thereby selectively inhibiting Gα13-mediated outside-in signaling and platelet aggregation while preserving talin–β3 interactions—a mechanism essential for αIIbβ3-dependent primary hemostasis. Consistent with its ability to preserve talin function in human clot formation and contractile primary hemostasis, KGDRR demonstrated an excellent safety profile in swine models and in assessments using the clinically validated ROTEM tool. These findings further reinforce KGDRR’s minimal hemorrhagic effects, highlighting its potential as a safer alternative to currently available αIIbβ3 antagonists.

In contrast, partial agonists, particularly eptifibatide, contain tryptophan side chains that point inward toward the RGD loop center, positioned away from the Tyr122 side chain of the βA domain. This structural arrangement prevents the formation of the critical central cation-π interaction, leading to weakened integrin stabilization. Since partial agonist-induced conformational changes affect both extracellular and intracellular domains, we hypothesize that their allosteric interactions with the integrin headpiece disrupt cytoplasmic leg interactions, preventing the maintenance of the low-affinity state [22]. As a result, partial agonists induce an open, active αIIbβ3 conformation, leading to integrin activation. Consistent with these findings, partial agonists such as eptifibatide and rhodostomin increase LIBS-specific mAb AP5 binding to platelets, demonstrating their ability to induce integrin conformational shifts and weaken talin binding to β3. Given talin’s crucial role in primary hemostasis and wound healing [17,18], partial agonists may effectively inhibit thrombosis but simultaneously impair hemostasis. These findings underscore the critical role of Arg55 in stabilizing αIIbβ3 in its inactive conformation, effectively minimizing bleeding risk while preserving essential hemostatic function.

The bleeding risks associated with αIIbβ3 antagonists significantly limit their clinical application, restricting both therapeutic dosing and overall effectiveness [14]. To assess the safety margin of KGDRR, we administered high doses of both TMV-7 and KGDRR. Unlike TMV-7 at high concentrations, which not only inhibited thrombin-stimulated clot retraction—a key αIIbβ3-dependent hemostatic process—but also led to a reduction in the coagulation index indicative of a hypocoagulation state, KGDRR preserved normal clot retraction function and hemostatic function. Additionally, even at elevated concentrations, KGDRR did not induce immune-mediated platelet clearance or prolong bleeding times in vivo. These results confirm that KGDRR exhibits a significantly broader safety margin than TMV-7, supporting its potential as a safer and more viable therapeutic option for clinical antithrombotic therapy.

To our knowledge, this is the first report of an R(K)GD-based pure αIIbβ3 antagonist derived from a snake venom disintegrin that exhibits a specific cation-π interaction with the integrin β3 domain. This interaction leads to selective inhibition of platelet aggregation and thrombus formation, while preserving integrin-dependent hemostatic processes. The key advantages of KGDRR over existing αIIbβ3 antagonists include: 1. Exceptional safety profile—KGDRR significantly reduces the risk of thrombocytopenia and bleeding, which are commonly associated with eptifibatide and tirofiban. 2. Pure αIIbβ3 antagonism—Unlike partial agonists, KGDRR prevents integrin conformational changes and LIBS exposure, thereby maintaining platelet stability. 3. Preservation of hemostatic function—KGDRR maintains clot retraction, ensuring physiological hemostasis while effectively preventing thrombosis.

Recently, Hr10, a pure orthosteric αIIbβ3 inhibitor, was reported to exhibit potent antithrombotic activity while preserving hemostasis [23]. KGDRR shares key characteristics with Hr10, including: 1. Pure αIIbβ3 antagonism—KGDRR effectively prevents conformational changes and LIBS exposure, reducing the risk of unintended platelet activation. 2. Stabilization of hemostatic function—KGDRR preserves clot retraction and contractile primary hemostasis, ensuring normal blood clot formation while inhibiting thrombosis. Additionally, KGDRR exhibits a stable pharmacokinetic profile when administered via intramuscular injection, offering greater convenience for pre-hospital emergency treatment and potentially reducing overall healthcare costs compared to standard intravenous infusions. However, further clinical investigations are necessary to establish the full therapeutic potential of KGDRR as a pure αIIbβ3 antagonist.

This study provides novel insights into the molecular interactions between disintegrins and αIIbβ3 and reveals the intrinsic mechanisms of αIIbβ3-talin and αIIbβ3-Gα13 signaling in physiological processes. These findings serve as a foundation for the development of next generation R(K)GD-based pure integrin β3 antagonists with minimized bleeding risks and improved therapeutic efficacy, paving the way for safer and more effective antithrombotic therapies.

## 4. Materials and Methods

Further details can be found in the Supplementary Methods and Materials.

### 4.1. The Expression of TMV-7 and Its Mutants in P. pastoris and Purification

The expression of TMV-7, ten TMV-7 mutants (R50K/N54W/P55N, R50K/N54W/P55R/G71R, R50K/N54F, N54F/P55R/G71R, R50K, P55R/G71R, N54A/P55R/G71R, R50K/N54R/G71R, N54R/G71R, N54R/P55R/G71R, R50K/N54R/P55R/G71R), and rhodostomin mutant (P50A/M54R/P55R) in *P. pastoris* was accomplished by following previously described protocols [24,25]. The expression kit and the yeast transfer vector, pPICZαA, were purchased from Invitrogen. The wild-type construct was used to produce the mutations using overlap extension PCR. The construct was transformed into the Pichia strain, X33, using a *Pichia* EasyComp kit from Invitrogen. We picked the highest TMV-7 and rhodostomin protein expression clone from a number of clones with multicopies of TMV-7 and rhodostomin gene insertion, respectively. The recombinant TMV-7, TMV-7 mutants, and rhodostomin mutant produced in *P. pastoris* were further purified by reversed-phase C_18_ HPLC with a gradient of 15–18% acetonitrile. The purification of recombinant TMV-7, TMV-7 mutants, and rhodostomin mutant was greater than 95% pure as judged by tricine-sodium dodecyl sulfate-polyacrylamide gel electrophoresis.

### 4.2. Definition of RR Mutant

We conducted a series of mutations on the TMV-7 sequence, focusing primarily on its RGD loop and C-terminal domain. An alanine scanning mutagenesis of the RGD loop revealed that the N54A (^50^ARGDAR^55^) mutation increases the safety index by 160-fold, indicating that Arg55 is crucial for minimizing bleeding risk. Building on these results, we further mutated ^50^ARGDAR^55^ to ^50^ARGDRR^55^ to enhance antiplatelet potency. Additionally, we performed an arginine-to-lysine substitution (R51K) to improve the specificity for the α_IIb_β_3_ receptor. In subsequent experiments, the resulting ^50^AKGDRR^55^ mutant was used as a candidate antithrombotic drug. Here, RR denotes the specific mutant variant of KGDRR under investigation.

### 4.3. Safety Index Calculation

The “safety index” was defined as the ratio between the lowest concentrations of disintegrin to activate platelets in the presence of mAb AP2 and the IC_50_ (μg/mL) for collagen-induced platelet aggregation. The mAb AP2, an IgG1 subclass inhibitory mAb raised against conformation-dependent epitopes, was used to mimic a drug-dependent antibody (DDAb) for predicting the adverse reaction of thrombocytopenia in the administration of antithrombotic agents [5].

Safety Index = the lowest concentration of disintegrin to activate platelet (combining with 4 μg/mL AP2)/IC_50_ of disintegrin on collagen-induced platelet aggregation.

### 4.4. Priming Assay

Priming assay was performed as described previously, with minor modifications [15,26]. Washed platelets in HEPES-modified Tyrode’s buffer were treated with the eptifibatide, TMV-2, TMV-7, or KGDRR for 30 min at RT, and then the platelets were fixed with 1% paraformaldehyde. After quenching the paraformaldehyde with glycine and washing, fluorescent fibrinogen (200 μg/mL) was added for 30 min at 37 °C, and then bound fluorescent fibrinogen was detected by flow cytometry.

### 4.5. Induction of Ligand-Induced Binding Site (LIBS)

Human PS were washed with HEPES-modified Tyrode’s buffer containing 1 μmol l^−1^ PGE_1_ (prostaglandin E_1_) and resuspended in modified Tyrode’s buffer containing 1 mmol/L MgCl_2_ and 2 mmol/L CaCl_2_. After incubating washed platelets with PBS (Ctl), eptifibatide (Ept, 1 μg/mL), TMV-2 (1 μg/mL), TMV-7 (1 μg/mL) or RR (0.5 μg/mL) for 30 min, the platelets were probed by 20 μg/mL mAb AP5 raised against LIBS. The expression of AP5 binding to α_IIb_β_3_ was analyzed by flow cytometry using FITC-conjugated anti-IgG mAb as a secondary antibody.

### 4.6. Rotational Thromboelastometry (ROTEM)

Blood was collected from the healthy donor. The effect of agents on hemostatic function was assessed using the ROTEM^®^ delta system with software 2.1-US (Haemoscope Corporation, Niles, IL, USA). ROTEM tests are started by re-calcification and accelerated by adding an activator of the intrinsic (e.g., INTEM; ellagic acid/phospholipid activation) or extrinsic (e.g., EXTEM; tissue factor activation) coagulation pathway. In this study, we monitor the coagulation process via the intrinsic pathway in the presence of agent administration. The changes in strength and elasticity in the clot provide information about how well the blood can perform hemostasis and how well or poorly different factors contribute to clot formation. For ROTEM, variables were taken as a representation of hemostasis: the clotting time (CT), the clot formation time (CFT), the α angle, and the maximum clot firmness (MCF). The CT value represents the time from initiation to initial fibrin formation. The CFT value is the time from the end of CT until the clot reaches 20 mm, representing the speed of clot formation. The α angle represents the speed at which fibrin build-up and cross-linking occur (clot strengthening) and hence assesses the rate of clot formation. The MCF is a reflection of clot strength. The CT, CFT, α angle, and MCF variables can also be incorporated into a coagulation index (CI) as defined by the equation: CI = −0.6516CT − 0.3772CFT + 0.1224MCF + 0.0759α − 7.7922. The CI functions as an overall assessment of coagulation, with values less than −3.0 said to be a hypocoagulable state, and values over +3.0 said to be a hypercoagulable state.

### 4.7. Data Analysis

The statistical significance of the results was analyzed using the GraphPad Prism program, version 6.0. Statistical significance was assessed by one-way ANOVA, followed by Dunnett’s multiple comparisons test as appropriate. *p* values < 0.05 were considered statistically significant.

## 5. Conclusions

This study identifies KGDRR as a novel and optimized αIIbβ3 antagonist with potent antithrombotic activity and a significantly improved safety profile. Structure–activity relationship and molecular docking analyses revealed a critical cation–π interaction between Arg55 in the engineered KGD loop and Tyr122 of the integrin β3 subunit, stabilizing αIIbβ3 in an unliganded-closed conformation. This structural feature selectively inhibits ligand binding and Gα13-mediated outside-in signaling while preserving talin-mediated interactions essential for hemostasis. In contrast to existing αIIbβ3 antagonists, KGDRR does not induce integrin activation, LIBS exposure, or thrombocytopenia. Its efficacy and safety were validated in murine and swine models, including the use of clinically relevant ROTEM analysis, which confirmed preserved clot retraction and minimal bleeding risk. Additionally, KGDRR demonstrated an extended functional half-life compared to eptifibatide. In summary, KGDRR represents a first-in-class, KGD-based αIIbβ3 antagonist that selectively inhibits thrombus propagation while preserving physiological hemostasis, supporting its promise as a next-generation antithrombotic therapy.

## Figures and Tables

**Table 1:** Comparison of IC_50_ in collagen (10 μg/mL)-induced human platelet aggregation, safety index, and tail-bleeding time in mice treated with clinical antithrombotic agents or mutated αIIbβ_3_ antagonists. Rn, disintegrin rhodostomin. For αIIbβ_3_ antagonists derived from TMV-7 or rhodostomin (Rn), mutated residues within the 50–55 RGD loop are underlined. All amino acid sequences are shown in single-letter code. IC_50_ was determined from in vitro inhibition of collagen-induced platelet aggregation in mouse platelet-rich plasma (PRP), as shown in the figure (panel A) in Section 2.7. The safety index was defined as described in the Methods section. Tail bleeding time was assessed after intravenous administration at 2×, 10×, or 20× the IC_50_ dose of each agent. Statistical significance was determined by comparison with the control group (62.78 s, *n* = 32). Data for the tested agents are also presented in the figure (panel E) in Section 2.7. Detailed methods for IC_50_ determination and tail bleeding time assessment are provided in the Supplementary Methods. Statistical significance was analyzed using Dunnett’s test (n.s., not significant; * *p* < 0.05; ** *p* < 0.01; *** *p* < 0.001).

Sequence of Antithrombotic Agents		IC_50_	Safety	Tail-Bleeding Time (sec)		
(^41^Linker^45^—^50^RGD Loop^55^—^68^C-Terminal Domain)		(nM)	Index	Dosage: Twice IC_50_	Dosage: 10 × Times IC_50_	Dosage: 20 × Times IC_50_
Eptifibatide		625.00	5.00	538.60 ***	1341.20 ***	
Tirofiban		85.11	4.00	480.67 ***	1443.60 ***	
Abciximab		105.02	4.25	373.38 ***	580.80 ***	
Non-RGD small molecule RUC-2		62.50	25.50	66.33 n.s.		
TMV-2: KKKGT-ARGDWN-PRNGLYG		41.65	6.25	353.70 **	1152.60 ***	
TMV-7: KKKRT-ARGDNP-PRNGLYG		58.41	23.40	76.50 n.s.	121.00 n.s.	188.00 *
TMV-7 mutant	KKKRT-ARGDWN-PRNGLYG	48.52	4.00	834.00 ***	725.80 ***	
	KKKRT-ARGDFP-PRNGLYG	68.75	7.00	417.50 ***	599.10 **	
	KKKRT-ARGDWR-PRNRLYG	32.27	277.89	66.00 n.s.	72.50 n.s.	
	KKKRT-ARGDFR-PRNRLYG	48.39	230.89	68.20 n.s.	85.00 n.s.	
	KKKRT-ARGDNR-PRNRLYG	44.05	268.82	70.20 n.s.	73.20 n.s.	
	KKKRT-ARGDAR-PRNRLYG	34.42	>3640.33	68.20 n.s.	86.00 n.s.	
	KKKRT-ARGDRR-PRNRLYG	31.41	>3709.20	71.40 n.s.	73.00 n.s.	81.60 n.s.
	KKKRT-AKGDRR-PRNRLYG	30.08	>3365.87	63.00 n.s.	61.50 n.s.	82.17 n.s.
Rn:	SRAGK-PRGDMP-PRYHRR	55.31	3.20	890.34 ***	1800.00 ***	
Rn mutant:	SRAGK-ARGDRR-PRYHRR	39.07	>3403.68	60.50 n.s.	63.50 n.s.	78.35 n.s.

**Table 2:** Statistical analysis of integrin α_IIb_β_3_–TMV7 and α_IIb_β_3_–KGDRR docking results obtained from HADDOCK webserver.

Parameters	Integrin α_IIb_β_3_	Integrin α_IIb_β_3_
	KGDRR	TMV-7
Electrostatic energy (kcal/mol)	−826.1 ± 76.7	−774.1 ± 42.8
Van der Waals energy (kcal/mol)	−22.6 ± 9.7	−20.1 ± 4.7
Restraints violation energy (kcal/mol)	2.2 ± 0.5	0.7 ± 0.1
Cluster size	200	143
Buried Surface Area (Å^2^)	1887.7 ± 70.0	1775.6 ± 43.7
RMSD (Å)	0.7 ± 0.5	0.8 ± 0.5

**Table 3:** The IC_50_ of KGDRR and Rhodostomin-RGDRR mutant (Rn-RR) in human PRP and washed human platelets. PRP, platelet-rich plasma; PS, washed platelet suspension; ADP, adenosine 5’-diphosphate. Data are mean (*n* = 5). N/A, not applicable.

Disintegrin	KGDRR (nM)		Rn-RR (nM)	
Inducer	PRP	PRP	PS	PS
ADP (20 μM)	21.46	N/A	35.60	N/A
Thrombin (0.1 U)	N/A	19.81	N/A	32.90
Collagen (10 μg/mL)	30.08	24.87	58.41	41.90
U46619 (1 μM)	30.62	19.28	52.30	31.00

## Data Availability

The data supporting this study’s findings are available from the corresponding author upon reasonable request. Some data may not be made available because of privacy or ethical restrictions.

## Supplementary Materials

The supporting information can be downloaded at: https://www.mdpi.com/article/10.3390/ijms26104530/s1. References [6,11,24,25,27,28,29,30,31,32,33,34] are cited in the supplementary materials.

## Abbreviations

CI	coagulation index
LIBS	ligand-induced binding site
mAb	monoclonal antibody
PCI	percutaneous coronary interventions
PRP	platelet-rich plasma
PS	platelet suspension
RGD	Arg-Gly-Asp
ROTEM	rotational-thromboelastometry
SAR	Structure–activity relationship
TMV	Trimeresurus mucrosquamatus venom
