# Supplementary material for: A Novel KGD-Based αIIbβ3 Antagonist Prevents Arterial Thrombosis While Preserving Hemostasis and Avoiding Thrombocytopenia
Source: Int J Mol Sci. 2025 May 9;26(10):4530. doi: 10.3390/ijms26104530 (PMC12111684; doi:10.3390/ijms26104530)
Supplement: Supplementary file 1 [file ijms-26-04530-s001.zip › ijms-3621327-supplementary.pdf]

A Novel KGD-Based αIIbβ3 Antagonist Prevents Arterial Thrombosis While Preserving Hemostasis and Avoiding Thrombocytopenia

Yu-Ju Kuo[1], Ching-Hu Chung[2], Chun-Chao Chen[1,3], Ju-Chi Liu[1,3], Kuan-Rau Chiou[1,3], Joen-Rong Sheu[4], Woei-Jer Chuang[5,*] and Tur-Fu Huang[2,6,*]

[1]Division of Cardiology, Department of Internal Medicine, Shuang Ho Hospital, Taipei Medical University, Taipei 23561, Taiwan; 23580@s.tmu.edu.tw (Y.-J.K.); b101092035@tmu.edu.tw (C.-C.C.); liumdcv@tmu.edu.tw (J.-C.L.); krchiou@hotmail.com (K.-R.C.)

[2]Department of Medicine, Mackay Medical College, New Taipei City 252, Taiwan; chchung@mmc.edu.tw

[3]Division of Cardiology, Department of Internal Medicine, School of Medicine, College of Medicine, Taipei Medical University, Taipei 23561, Taiwan

[4]Graduate Institute of Medical Sciences, College of Medicine, Taipei Medical University, Taipei 11031, Taiwan; sheujr@tmu.edu.tw

[5]Department of Biochemistry, National Cheng Kung University Medical College, Tainan 701, Taiwan

[6]Graduate Institute of Pharmacology, College of Medicine, National Taiwan University, Taipei 100233, Taiwan

***Correspondence:** wjcnmr@mail.ncku.edu.tw (W.-J.C.); turfu@ntu.edu.tw (T.-F.H.)

Data availability statement: The data supporting this study's findings are available from the corresponding author upon reasonable request.

Supplementary Methods and Materials.

Supplementary Materials

The expression of TMV-7 and its mutants in P. pastoris and purification

The expression of TMV-7, ten TMV-7 mutants (R50K/N54W/P55N, R50K/N54W/P55R/G71R, R50K/N54F, N54F/P55R/G71R, R50K, P55R/G71R, N54A/P55R/G71R, R50K/N54R/G71R, N54R/G71R, N54R/P55R/G71R, R50K/N54R/P55R/G71R), and rhodostomin mutant (P50A/M54R/P55R) in P. pastoris was accomplished by following protocols previously described[1, 2]. The expression kit and the yeast transfer vector, pPICZαA, were purchased from Invitrogen. The wild-type construct was used to produce the mutations using overlap extension PCR. The construct was transformed into the Pichia strain, X33, using a Pichia EasyComp kit from Invitrogen. We picked the highest TMV-7 and rhodostomin protein expression clone from a number of clones with multicopies of TMV-7 and rhodostomin gene insertion, respectively. The recombinant TMV-7, TMV-7 mutants, and rhodostomin mutant produced in P. pastoris were further purified by reversed-phase C_{18} HPLC with a gradient of 15–18% acetonitrile. The purification of recombinant TMV-7, TMV-7 mutants, and rhodostomin mutant was greater than 95% pure as judged by tricine-sodium dodecyl sulfate-polyacrylamide gel electrophoresis (SDS-PAGE).

Preparation of human platelet-rich plasma and platelet suspensions, and aggregation assay

Blood samples were freshly collected from healthy adult volunteers who had not taken any medications or supplements known to interfere with platelet function (e.g., aspirin, NSAIDs, or anticoagulants) within two weeks prior to donation. All participants provided written informed consent, and the study was approved by the Ethics Committee and Institutional Review Board of National Taiwan University Hospital, Taiwan (IRB No. 201406057RINB). Health status and eligibility were assessed through a standardized pre-donation screening questionnaire administered by a licensed medical technologist. This included evaluation of recent medication use, surgical history, and bleeding tendencies. Only donors who met the criteria for good health and absence of interfering agents were included. Human platelet-rich plasma (PRP) and platelet suspensions (PS)

were prepared according to the method described previously [3]. Platelet aggregation was measured with a Lumi-Aggregometer (Payton Scientific, Buffalo, NY, USA) under continuous stirring at 900 rpm. Human PRP or PS was added to the silicon-coated cuvette and incubated at 37°C with an appropriate amount of Tyrode's buffer. Test agents were added 3 minutes prior to the addition of an agonist.

Co-immunoprecipitation

Washed human or mouse platelets were pre-incubated with agents three minutes prior to the addition of thrombin (0.1 U/mL), and then solubilized in modified RIPA Buffer (50 mM Tris, pH 7.4, 10 mM $MgCl_2$, 150 mM NaCl, 1% NP-40, 1 mM sodium orthovanadate, 1 mM NaF) at various time points. Cell lysates were then immunoprecipitated with anti-integrin β_3 for 1 hr and further incubated with Protein A/G sepharose beads (Santa Cruz). After incubation of Protein A/G sepharose beads at 4°C overnight, beads were centrifuged down and washed for five times with ice-cold PBS. Immunoprecipitates were analyzed by SDS-PAGE and Western blots with antibodies against integrin β_3, $G\alpha_{13}$, and talin as described previously [4].

All primary antibodies used in this study were diluted 1000 times for western blotting. The following antibodies were used in this study: Anti-talin (#sc7534), anti-$G\alpha_{13}$ (#sc410), anti-integrin β_3 (#sc6627), and anti-c-Src (#sc18) antibodies were purchased from Santa Cruz Biotechnology, Inc. (Santa Cruz, CA, USA); anti-RhoA and anti-integrin β_3 (#CBL479) antibodies were from Millipore.

Clot retraction

Clot retraction was measured in vitro using human PRP according to the previously described protocol [5, 6], with minor modifications. Human PRP was prepared as described above. Filling glass tubes with 200 µL PRP, 5 µL RBC (used to color the clot) and an appropriate amount of Tyrode's solution and equilibrating at 37°C for 1 min. Then, PBS, abciximab, TMV-2, TMV-7, KGDRR, or RUC-2 was added to each tube 3 min before the addition of thrombin. After adding thrombin, immediately add a sealed glass pipette to the center of the tube and place all the tubes at where it can be undisturbed for several hours. To observe the kinetics of

clot retraction, photographs taken at time 0 and every 15 min until 120 min. Results from clot retraction are assessed numerically by the size of the clot and % retraction. The clot surface area was quantitated using a Java plugin for the Image J software (National Institute of Mental Health, Bethesda, Maryland, USA). The % retraction was measured by the volume of serum (test)/volume of serum (control).

Platelet adhesion assay

As described previously[7], before plating to well microliter plates coated with fibrinogen (50 μg/mL), washed platelets were labeled with fluorescent dye BCECF-AM and then pre-incubated with PBS or antithrombotics for 30 min at 37 ºC. After 1 hr incubation at 37 ºC, adherent platelets were photographed using a photoMicroGraphic Digitize integrate System (MGDS; Total integral Technology Co., Ltd., Taipei, Taiwan). Adhesion of platelets was quantified as the percentage of fluorescence intensity of control platelets.

Platelet spreading

For the spreading experiments, coverslips were coated with fibrinogen overnight at 4°C. After twice washing with PBS, the coverslips were blocked with 1% BSA in modified Tyrode's buffer for 1 hr at RT. BSA-coated coverslips were prepared for negative control. Washed platelets suspended in modified Tyrode's buffer (2×10^7/mL) were treated with thrombin and then promptly seeded on the coverslips for 45 minutes at 37°C in the presence or absence of TMV-2, TMV-7, or KGDRR. After removal of unbound platelets, coverslips were washed with PBS, and then platelets were fixed by 4% paraformaldehyde for 30 min at RT, permeabilized by 0.1% Triton X-100 for 20 min, and stained by primary antibody anti-β_3 (followed by FITC-conjugated anti-goat secondary antibody) and Alexa Fluor 546-conjugated phalloidin (Invitrogen) for 2 hr. Platelets were observed using a Zeiss A1 AxioImager fluorescence microscope equipped with 100×, oil immersion lens, and then recorded using a Zeiss AxioCam ICC 3 color camera and AxioVision image capture software. To compute the surface area of spreading platelets, images were manually outlined and quantitated by determining the number of pixels within each outline using a Java plugin for the Image J software (National

Institute of Mental Health, Bethesda, Maryland, USA).

Animal Preparation

The male ICR and FcγRIIa-transgenic mice (weighing 24-30 g) and Lanyu-200 pigs (weighing 30-40 kg) were used in all studies. All surgical procedures on animals are performed under anesthesia. Mice were anesthetized with sodium pentobarbital (50 mg/kg, intraperitoneal). Pigs were anesthetized with Telazol (Tiletamine with zolazepam, intramuscular injection, 6-8.8 mg/kg) followed by inhalation of Isoflurane (1-3%) for maintenance of anesthesia. All experiments with animals were performed in accordance with the ARRIVE Guidelines and followed the recommendations of the NIH Guide for the Care and Use of Laboratory Animals. All procedures were approved by the Laboratory Animal Care and Use Committee (20150503) of the College of Medicine, National Taiwan University.

In vitro and ex vivo mouse platelet aggregation

Male ICR was anesthetized with sodium pentobarbital (50 mg/kg, intraperitoneal). For in vitro mouse platelet aggregation assay, blood samples were collected by intracardiac puncture. PRP was obtained by centrifuging the blood sample at 200g for 4 min and preincubated with saline, eptifibatide, TMV-2, TMV-7 or KGDRR for 3 min, and then treated with 10 μg/mL collagen (Sigma-Aldrich Chemical Co.). For ex vivo mouse platelet aggregation assay, mice were treated intravenously with saline, eptifibatide, TMV-2, TMV-7 or KGDRR. Blood samples were collected at 5 min by intracardiac puncture. Mouse PRP was obtained by centrifuging the blood sample at 200g for 4 min, and then treated with collagen. Platelet aggregation was measured as described above.

Fluorescent dye-induced mesenteric venous thrombosis model

Fluorescent dye-induced platelet thrombus formation in mesenteric microvessels of male ICR was performed as described previously[8] with some modifications. In brief, mice were injected intravenously with fluorescein sodium before administration of saline, eptifibatide, TMV-7, or KGDRR through a lateral tail vein,

and the microvascular bed was irradiated by filtered light from a 100-W mercury lamp with a filter (B-2A, Nikon, Tokyo, Japan) and dichromic mirror (DM 510, Nikon) in the epi-illumination system. The thrombus formation was observed using an Olympus IX71 inverted phase/fluorescence microscopes equipped with 10× objective lenses, and then recorded using an Olympus DP71 digital microscope camera (Olympus America Inc.) and version 4.6 of the SPOT software (Micro Video Instruments, Inc.).

Ferric chloride ($FeCl_3$)-induced arterial thrombosis model

Male ICR mice were anesthetized, and the right common carotid artery was exposed. Intravenous injection of saline, eptifibatide, TMV-7 or KGDRR was performed 15 min before experimentation. Mice were intravenously administered with agents for 5 min, $FeCl_3$ injury was induced with a filter paper disc (diameter, 2 mm) soaked with 7.5% $FeCl_3$ and observed by a miniature Doppler flow probe (Model 0.5VB; Transonic Systems, Ithaca, NY) placed around the artery. After 3 min exposure, the filter paper was then removed, and thrombus formation in carotid artery was monitored via the blood flow rate until complete occlusion or for 80 min. A 2-mm section of the carotid artery within the site of injury was taken as a pathologic sample. We performed the histological examination as described previously[9]. The section of the carotid artery was observed using an Olympus CX31 fixed specification binocular biological microscope equipped with 10× objective lenses, and then recorded using an Olympus DP12 digital microscope camera (Olympus America Inc.).

Hemostasis in a FcγRIIa-transgenic mouse model

Mice transgenic for human FcγRIIa (B6; SJL-Tg [FCGR2A]11Mkz/J) were generated in the laboratory of SEM[10] and obtained from The Jackson Laboratory (Bar Harbor, ME). Mouse genotypes were confirmed by The Jackson Laboratory-defined PCR protocol and used for experiments when 8~20 weeks old.

<u>Platelet counts in FcγRIIa-transgenic mice</u>

The FcγRIIa-transgenic mice were intravenously treated with eptifibatide (Ept, 0.25 mg/kg), TMV-2 (0.5 mg/kg), TMV-7 (0.5 mg/kg), or RR (0.25 and 2.5 mg/kg), and

then whole blood (100 μL) was collected by puncture of the retro-orbital sinus of anesthetized mice using heparinized hematocrit tubes. After blood was anticoagulated with sodium citrate for 5 min, the platelet numbers of the whole blood samples were counted by a Sysmex cell counter (Chuo-Ku Kobe, Japan).

Tail bleeding time in FcγRIIa-transgenic mice

Analysis of tail bleeding time was performed as described previously[11]. Briefly, the FcγRIIa-transgenic mice were intravenously injected through a lateral tail vein with saline, eptifibatide, TMV-2, TMV-7, or KGDRR for 5 min, and then the mouse was placed in a tube holder with its tail protruding and 2 mm segment from the distal tail was severed. The amputated tail was immediately immersed in isotonic saline at 37°C. Bleeding time was recorded for a maximum of 15 min. Endpoints defined as no evidence of mouse tail-rebleeding for 1 min.

Anti-platelet activity and hemostatic function in a pig model

The Lanyu-200 pigs were used in this study. All surgical procedures on pigs are performed under anesthesia. Pigs were anesthetized with Telazol (Tiletamine with zolazepam, intramuscular injection, 6-8.8 mg/kg) followed by inhalation of Isoflurane (1-3%) for maintenance of anesthesia.

In vitro and ex vivo pig platelet aggregation

An indwelling 14-gauge central venous catheter was inserted into the right external jugular vein for collecting blood samples. The collected blood samples at indicated time intervals (i.e; 0, 5, 30 and 60 min) were tested for platelet counts and platelet aggregation function. Pig PRP was obtained by centrifuging the blood sample at 1800 rpm for 9 min, and preincubated with eptifibatide or KGDRR for 3 min, and then treated with 10 μg ml^{-1} collagen. Platelet aggregation was measured as described above.

Bleeding time and platelet counts in pigs

A pig ear vein was cannulated for application of the tested agents including KGDRR and Eptifibatide. Bleeding time was measured before and administration of the tested agents after 5, 30 and 60 min. The bleeding time was defined as the time from incision to a cessation of bleeding at a shaved inner site on the ear using a standard cutting device (Quick -Heel Lancet, BD, USA). Shed blood was carefully

removed at the exactly 15-sec interval with a filter paper. At each time point, the means of independent triplicate measurements was recorded as the bleeding time. If the bleeding time exceeded 900 sec, the bleeding time was recorded as 900 sec and further bleeding was stopped. Four months after the platelet aggregation and hemostatic, the Lanyu pigs on the study were sacrificed by intravenous injection of KCl (2 mmol/kg) after anesthetization with Telazol (Tiletamine with zolazepam, intramuscular injection, 18-26.4 mg/kg).

Molecular docking of TMV-7 and KGDRR into integrin $\alpha_{IIb}\beta_3$

The interaction restraints were derived from the X-ray structure of integrin $\alpha_{IIb}\beta_3$ in complex with a GRGDSP peptide (PDB code 3ZE2)[12] by using CCP4i software (http://structure.usc.edu/ccp4/). The defined distance threshold was 4 Å, and the interaction restraints between the RGD motif and integrin were used for the calculation. The first run input restraints between the G52 and D53 residues and integrin $\alpha_{IIb}\beta_3$ were 12, and 45, respectively. These input restraints were the contacts between the G52 residue and residue Tyr190 of α_{IIb} and residues Arg216 and Ala218 of β_3; and between the D53 residue and Mn^{2+} of the MIDAS of β_3 and residues Ser121, Tyr122, Ser123, Arg214, Asn215, Arg216, Asp217, Ala218, and Glu220. An additional 0.5-Å distance was added to the upper and lower limit in the interaction restraints. These restraints were used to perform the standard HADDOCK protocol for protein docking with minor modifications.

References

1. Guo RT, Chou LJ, Chen YC, Chen CY, Pari K, Jen CJ, et al. Expression in Pichia pastoris and characterization by circular dichroism and NMR of rhodostomin. Proteins. 2001; 43: 499-508.
2. Shiu JH, Chen CY, Chen YC, Chang YT, Chang YS, Huang CH, et al. Effect of P to A mutation of the N-terminal residue adjacent to the Rgd motif on rhodostomin: importance of dynamics in integrin recognition. PLoS One. 2012; 7: e28833.
3. Huang TF, Chang CH, Ho PL, Chung CH. FcgammaRII mediates platelet aggregation caused by disintegrins and GPIIb/IIIa monoclonal antibody, AP2. Exp Hematol. 2008; 36: 1704-13.

4. Shen B, Zhao X, O'Brien KA, Stojanovic-Terpo A, Delaney MK, Kim K, et al. A directional switch of integrin signalling and a new anti-thrombotic strategy. Nature. 2013; 503: 131-5.
5. Huang TF, Wang WJ, Teng CM, Liu CS, Ouyang C. Purification and characterization of an antiplatelet peptide, arietin, from Bitis arietans venom. Biochim Biophys Acta. 1991; 1074: 136-43.
6. Tucker KL, Sage T, Gibbins JM. Clot retraction. Methods Mol Biol. 2012; 788: 101-7.
7. Hsu CC, Wu WB, Huang TF. A snake venom metalloproteinase, kistomin, cleaves platelet glycoprotein VI and impairs platelet functions. J Thromb Haemost. 2008; 6: 1578-85.
8. Chang MC, Huang TF. In-Vivo Effect of a Thrombin-Like Enzyme on Platelet Plug Formation Induced in Mesenteric Microvessels of Mice. Thrombosis Research. 1994; 73: 31-8.
9. Kwon I, Hong SY, Kim YD, Nam HS, Kang S, Yang SH, et al. Thrombolytic Effects of the Snake Venom Disintegrin Saxatilin Determined by Novel Assessment Methods: A FeCl3-Induced Thrombosis Model in Mice. Plos One. 2013; 8.
10. McKenzie SE, Taylor SM, Malladi P, Yuhan H, Cassel DL, Chien P, et al. The role of the human Fc receptor Fc gamma RIIA in the immune clearance of platelets: A transgenic mouse model. Journal of immunology. 1999; 162: 4311-8.
11. Chang MC, Lin HK, Peng HC, Huang TF. Antithrombotic effect of crotalin, a platelet membrane glycoprotein Ib antagonist from venom of Crotalus atrox. Blood. 1998; 91: 1582-9.
12. Zhu J, Zhu J, Springer TA. Complete integrin headpiece opening in eight steps. J Cell Biol. 2013; 201: 1053-68.
